# No Effect of Long-Term Risedronate Use on Cartilage and Subchondral Bone in an Experimental Rabbit Model of Osteoarthritis

**DOI:** 10.3389/fvets.2020.576212

**Published:** 2020-11-02

**Authors:** Silvia Fernández-Martín, María Permuy, Mónica López-Peña, Fernando Muñoz, Antonio González-Cantalapiedra

**Affiliations:** Department of Anatomy, Animal Production and Veterinary Clinical Sciences, Veterinary Faculty, Universidad de Santiago de Compostela, Lugo, Spain

**Keywords:** bisphosphonate, bone, cartilage, osteoarthritis, risedronate, synovial membrane, subchondral bone

## Abstract

Osteoarthritis (OA) is the most prevalent degenerative joint disease in animals and humans. It is characterized by pain, articular cartilage damage and joint stiffness. It has been suggested that the status of the subchondral bone compartment plays an important role in the initiation and progression of OA. Bisphosphonates have been proposed as a potential disease-modifying treatment for OA, however their effectiveness is not yet clear. Twenty-four male adult New Zealand rabbits were used to evaluate the effects of risedronate on the subchondral bone quality and cartilage degradation in a long-term model of experimentally induced OA. Animals underwent an anterior cruciate ligament transection and partial medial meniscectomy or sham operation in only one knee, which was randomly chosen, using the contralateral as healthy control. Animals were divided into three groups (*n* = 8): untreated control group and sham surgery control group; both groups received only vehicle; and risedronate group, treated with 2.5 mg orally weekly for 24 weeks. Stifle joints were harvested and scanned using a high-resolution micro-CT to evaluate the subchondral plate and trabecular bone changes. The macroscopic evaluation and histological analysis were determined using an adapted Osteoarthritis Research Society International scoring scheme to assess the cartilage degeneration. The lateral and medial femoral condyle and tibial plateau were evaluated. Additionally, the histological synovial membrane assessment was carried out. Sample analysis showed that the experimental model induced osteoarthritic changes in the operated joints, whereas in sham-operated rabbits, almost no histological changes were observed on articular cartilage surfaces. In terms of macroscopic and histological analyses, risedronate-treated animals did not show improved cartilage health compared with untreated operated rabbits, but a slightly anti-inflammatory activity was observed in the synovial membrane. Risedronate administration showed a slight tendency to increase subchondral bone plate thickness in lateral compartments but, it did not show conservation of periarticular bone and was not be able to suppress the osteophyte formation. In conclusion, long-term risedronate use did not demonstrate a positive effect on reducing the cartilage damage, and failed to prevent the subchondral bone changes and osteophytogenesis in an experimental rabbit model of OA.

## Introduction

Osteoarthritis (OA) is a degenerative complex multifactorial disease process and the most common form of articular disorder in both human and veterinary medicine, affecting up to 15% of the human global population (1) and around 20% of the canine population over a year-old (2). It is usually characterized by pain, articular cartilage damage and joint stiffness. Although OA has traditionally been described as an articular cartilage alteration, this pathology involves all tissues in the synovial joint, hyaline cartilage, subchondral bone, meniscus, and periarticular soft tissues. In recent years, it has been suggested that the integrity of articular cartilage was tightly related with the periarticular bone tissue status (3–6). Moreover, it is currently believed that bone plays an important role during initiation and progression of OA (7–9). However, the exact relationship between cartilage, subchondral bone and other pathological changes is not clear yet and there is controversy about whether the subchondral bone changes follow, concur with, or precede the cartilage erosion in the pathogenesis of the disease (10). The changes observed in bone compartment during progression of OA include the thinning of the trabecular structure, an increase in the turnover of the subchondral bone, sclerosis of the subchondral plate, lesions in the bone marrow, and osteophyte formation (4, 5). Specifically, it has been suggested that the thickness of the subchondral bone plate may be related with the progression of OA (11) and an increase in the bone resorption may be in part responsible for the initial cartilage breakdown in stressful conditions (5).

Subchondral bone turnover depends on the osteoclastic activity, nevertheless the role of osteoclasts in the pathogenesis of OA still requires a great deal of attention (4). In that regard, several studies have analyzed the effect of antiresorptive substances on non-clinical OA, with the premise that these treatments would inhibit excessive subchondral bone turnover and consequently, reduce cartilage degradation (3, 12–14). Bisphosphonates (BPs) are powerful agents that inhibit bone loss and increase bone mineralization. Their main use is related to the treatment of pathologies with an increased bone turnover rate, such as osteoporosis and Paget's disease of bone. Currently, there is an increasing interest in their application in OA (7, 15, 16). Treatment with BPs seems to induce a decrease in the severity of OA scores, showing less cartilage pathology and bone changes in experimental animal models such as rats (3, 10, 17, 18), and rabbits (13, 19, 20). But it is still controversial whether these are effective in stopping the disease progression, because in some osteoarthritis preclinical research using guinea pigs (21), rats (14), and dogs (22), it was reported that the administration of BPs showed no effect on preventing the articular cartilage damage or even worsened the cartilage conditions in spite of greater subchondral bone quality. In the case of risedronate (RIS), which is one of the most studied, and potent BPs in animal models and humans, it was reported that administered in early stages of experimental OA, could alter the short-term progression by slowing down periarticular bone changes in rats (17), and rabbits (23, 24). Conversely, other publications described that RIS administration failed to prevent or correct the cartilage deterioration (12, 25, 26). Although RIS did not show an evident disease-modifying effect in clinical human trials, several studies described potential advantages in OA pathogenesis, such as improving some symptoms of knee OA, reducing cartilage degradation markers (15, 27), and preserving the subchondral and cancellous bone structure (28, 29).

Animal models are used to study the phases of the disease and to evaluate the possible clinical use of osteoarthritic drugs. Surgically-induced instability models have been studied in several animal species as an attempt to resemble traumatic human OA (30). An anterior cruciate ligament disruption or meniscal damage predisposes to secondary OA, as a result of joint instability (31). Consequently, this will cause mechanical changes in loading patterns with an associated rapid and severe cartilage erosion and subchondral bone alterations (30). Nowadays, rabbits are widely used as animal models of OA given that the characteristics of the cartilage lesions after surgical induction procedures are very similar to those observed in human disease, such as cartilage surface roughness which progresses to fissures, erosion, and even cartilage full-thickness ulceration (32, 33). Additionally, some anatomical similarities between rabbit and human knees are present, such as the existence of the patellar ligament, two menisci, and the suprapatellar synovial recess. However, it is important to be mindful of the differences in gait and the cartilage structure and composition when comparing rabbits and humans (33–35).

In relation to how to quantify the disease progression in animal models, histological and macroscopic evaluations are the gold standard techniques, allowing the direct visualization of articular cartilage and other non-osseous structures involved in the pathophysiology of OA (36, 37). In addition, new non-invasive, multi-modality imaging techniques offer a different and complete strategy to evaluate the bone microstructure in animal models of experimentally induced OA. Microfocal computed tomography (micro-CT) has proven to be an accurate tool to precisely measure changes in bone volume, density, and architecture (36).

In this manuscript we determined the extent of the changes that occur in large stages of OA and evaluate the effects of RIS administration on the joint structures by the use of high-resolution three-dimensional micro-CT, macroscopic evaluation, and histology assessments. We hypothesized that the administration of bisphosphonates would potentially be used as disease-modifying agents in OA, reducing the periarticular bone turnover and the trabecular bone microarchitecture changes. Actually, there are no studies that focused on the effect of risedronate on the articular cartilage and subchondral bone plate in a long-term model in rabbits.

The aim of this study was to determine whether risedronate therapy could slow down or prevent periarticular bone changes and hyaline cartilage degeneration in a rabbit post-traumatic model of knee osteoarthritis after 6 months of treatment.

## Materials and Methods

### Animals and Ethical Statement

For this study, twenty-four healthy skeletally mature male New Zealand rabbits (Granja San Bernardo, Navarra, Spain) of 6–7 months of age and mean weight of 5 Kg were used. All *in vivo* procedures were approved by the Ethical Committee of the University of Santiago de Compostela (Reference number: 01/16/LU-002) as a randomized controlled trial with two inter-subject controls for the comparison of one treatment option. Procedures were performed in accordance with Spanish and European Union regulations about care and use of research animals. In addition, this paper was written following the ARRIVE guidelines (38). The animals were maintained at the Animal Experimentation Facility of the University of Santiago de Compostela (Lugo, Spain) in enriched rabbit cages (R-suite, Tecniplast, Varese, Italy) under controlled light-dark cycle, humidity, and temperature. They had free access to food and tap water and all efforts were made to minimize animal pain and distress. Environmental enrichment included supply of previously autoclaved herb, fresh fruit, paper rolls, and wood sticks. All animals were monitored daily by a veterinarian accredited in laboratory animal science, who checked their health status and signs of discomfort. Body weight was measured at the beginning of the experiment, in the first month, and at the end of the experiment.

### OA Induction Procedure

To perform the surgical procedure, animals were firstly premedicated with a combination of medetomidine (50 μg/Kg IM, Domtor, Esteve, Barcelona, Spain), ketamine (25 mg/Kg IM, Imalgène 1000, Merial, Toulouse, France) and buprenorphine (0.03 mg/Kg IM, Buprex, RB Pharmaceuticals, Berkshire, UK). They were anesthetised using isoflurane general anesthesia (Inspiratory Fraction ISO 2.5–4%, Isova-vet, Schering-Plow, Madrid, Spain) using a facemask. Each animal received antibiotic prophylaxis with enrofloxacin (5 mg/Kg SC Ganadexil 5%, Invesa, Barcelona, Spain) and pain control with meloxicam (0.2 mg/Kg SC, Metacam, Boehringer Ingelheim España, Barcelona, Spain) for 5 days.

After 3 weeks of acclimatization, osteoarthritis was induced surgically by anterior cruciate ligament transection (ACLT) and partial medial meniscectomy, achieving the destabilization, and misalignment of the knee joint. Only one knee per animal was operated, using the contralateral joint as healthy control. This procedure was performed in sixteen animals. The remaining eight animals made up the sham operated control group, with a similar approach to the knee joint, but in this case the anterior cruciate ligament and medial meniscus were left intact. Surgical incision was closed in two layers using absorbable sutures. Post-operatively, animals were permitted free activity without joint immobilization.

### Risedronate Administration

**Figure 1:**
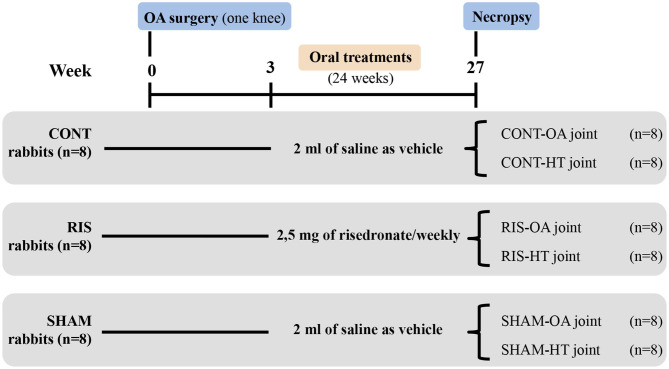
Experimental design. Rabbits were divided into three groups: (CONT) untreated control group, (RIS) risedronate treated group, and (SHAM) surgery control group. Operated joints constituted osteoarthritic groups (OA), while contralateral joints were used as healthy controls (HT).

Treatment began 3 weeks after surgery and was orally administered during 24 weeks. Animals were randomly assigned to one of the three groups (*n* = 8 each), according to a computer-generated randomization list. The first group received only saline as vehicle (NaCl 0.9%) and made up the control group (CONT). The second group received a 2.5 mg once-weekly dose of risedronate (RIS), whereas the third group received only vehicle and was the surgery control group (SHAM). In addition, operated joints made up the osteoarthritic groups (OA) and contralateral non-operated joints, the healthy groups (HT) (Figure 1).

The type of bisphosphonate and the dosage was selected based on the findings of a previous study from our laboratory (24). This dosage corresponded with the relative dose currently used in bisphosphonate treatment of osteoporosis in humans, and it is directly based on the clinical doses of 35 mg/week of RIS, assuming a 60 kg weight person, and considering 0.6% of the dose is bioavailable when administered orally (39).

### Necropsy and Samples Retrieval

After sedation with ketamine (25 mg/Kg IM, Imalgène 1000, Merial, Toulouse, France), and medetomidine (50 μg/Kg IM, Domtor, Esteve, Barcelona, Spain), animals were euthanized by a sodium pentobarbital injection in the lateral auricular vein (100 mg/Kg IV, Dolethal, Vétoquinol, Madrid, Spain). Immediately after euthanasia, stifle joints were harvested by sawing femora and tibias ~20 mm from the joint and dissected free of muscle and soft tissue. The specimens were preserved in 10% buffered formalin.

### Microfocal Computed Tomography

All specimens, containing proximal tibia and distal femur, were scanned before being processed for histology using a high-resolution micro-CT (Skyscan 1172, Bruker microCT NV, Kontich, Belgium). The X-ray source was set at 100 kV and 100 μA with a voxel size of 12.85 μm and an aluminum/copper filter (Al/Cu). The scanning was performed over a 360° rotation with image acquisition every 0.4°. Later scans were reconstructed using NRecon® software (Bruker microCT NV, Kontich, Belgium) using the algorithm described by Feldkamp (40). Reconstructed images were evaluated with CTAn software (Bruker microCT NV, Kontich, Belgium).

The following evaluations were performed:

#### Osteophyte Formation

The total volume (TV, mm^3^), the bone volume fraction (BV/TV, %) and the volumetric bone mineral density (vBMD, mg/cm^3^) in the femur were measured, using as volume of interest (VOI) a manual delineation of all the osteophytes.

#### Subchondral Bone Plate Thickness

Using a sagittal view of the lateral and medial compartments of both tibia and femur. Mean values were delimited and measured using the Image-Pro Premier 9.0 software (Media Cybernetics, Bethesda, MD, USA).

#### Trabecular Bone

The VOI selected included 200 slides of femur bone tissue and 100 slides of tibia and was set beginning at the closest point to the articular space where the trabecular bone was seen only surrounded by cortical bone. The trabecular bone parameters were obtained using the CTAn software and drawing a manual VOI that included only the trabecular bone. Threshold values for bone were finally set ranging between 55 and 255. vBMD values of trabecular bone were obtained comparing two phantoms of hydroxyapatite scanned in the same conditions as the samples. Standard morphometric parameters were determined to describe subchondral trabecular microstructure (41), including bone volume fraction (BV/TV, %), trabecular thickness (Tb.Th, mm), trabecular separation (Tb.Sp, mm), trabecular number (Tb.N, mm^−1^), and trabecular bone pattern factor (Tb.Pf, mm^−1^).

### Macroscopic Evaluation

The macroscopic evaluation of each sample was carried out using Osteoarthritis Research Society International (OARSI) scoring scheme (33). Once the joint cavity was opened, digital images were obtained using a stereo microscope coupled to a digital camera (SZX12, DP71; Olympus, Japan). An in depth description of the changes found around articular cartilage in both lateral and medial femoral condyles (LFC and MFC), lateral and medial tibial plateau (LTP and MTP), meniscus and osteophyte presence was performed. Changes observed were graded as follows: evaluation of cartilage ranging from 0 (smooth surface) to 4 (complete erosion of cartilage); changes observed in meniscus ranging from 0 (normal) to 5 (presence of complete fissures) and finally, the presence of osteophytes ranging from 0 (absent) to 3 (severe).

### Histological Assessment

Stifle joints were carefully dissected. Medial and lateral sections of the femoral condyles and tibia plateau were obtained using a band saw. Additionally, a synovial membrane fragment adjacent to the patellar ligament was obtained from each joint (42). Samples were decalcified (Osteodec, Bio-Optica, Milano, Italy), paraffin-embedded, and sectioned at 6 μm using a microtome (Leica RM 2255, Leica Biosystems, Wetzlar, Germany). The slides were stained with hematoxylin and eosin (H-E). All the sections were captured using a motorized stage light microscope and a PC-based image capture system (BX51, DP71, Olympus Corporation, Japan). The severity of OA lesions were graded histologically using an adapted scoring system from Osteoarthritis Research Society International (OARSI) (33, 43) (Table 1). Two independent and experienced observers blinded to the treatments performed the scoring.

**Table 1:** Histological gradation of the articular cartilage and synovial changes.

**Cartilage**	
**Severity of cartilage pathology characteristics**	
Normal volume, smooth surface with all zones intact	0
Surface undulations including fissures in surface/upper zone	1
Fissures to mid zone and/or erosion of surface/upper zone	2
Fissures to deep zone and/or erosion through mid zone	3
Full thickness loss of cartilage	4
**Severity of chondrocyte pathology characteristics**	
Normal	0
Loss of superficial cells or relative increased density with occasional clusters	1
Small clusters (2–4 cells) predominate	2
Large clusters (≥5 cells) predominate	3
Cell loss (necrosis/apoptosis) predominate	4
**Tidemark**	
Intact and distinct	0
Not consistent or distinct (loss and/or duplication)	1
Loss of Tidemark which is crossed by blood vessels	2
**Total**	10
**Synovial membrane**	
**Lining cell characteristics**	
1–2 layers of cells	0
3–6 layers of cells	1
>6 layers of cells	2
**Hyperplasia**	
No villous hyperplasia	0
Short villi	1
Finger-like hyperplasia	2
**Cell infiltration characteristics**	
No cellular infiltration	0
Mid to moderate inflammatory cell infiltrates including small lymphoid follicles	1
Marked, diffuse inflammatory cell infiltrates including large lymphoid follicles	2
**Total**	6

*Parameters evaluated in the gradation of histological changes in paraffin embedded samples*.

### Statistics

Sample size and analysis were conducted using SigmaPlot 12.5 software (Systat Software Inc., Chicago, IL, USA). All data were expressed as means ± standard deviations (SD). The normality of the data was assessed using the Shapiro-Wilk test. Levene's test was used to assess the equality of variances of normal variables and the statistical comparison was performed using ANOVA. *Post-hoc* analysis was carried out by applying the Holm-Sidak method. For non-normal variables, the statistical comparison was performed using the Kruskal-Wallis *H-*test and the *post-hoc* analysis using Dunn's test. Differences were considered significant at *p* < 0.05.

## Results

### Animals

All surgeries were performed without complications. One rabbit of the SHAM group died prematurely for unknown reasons. The rest of the animals tolerated the treatments successfully. There were no differences in the final body weight between groups, demonstrating that risedronate administration do not produce adverse effects on the growth of the animals. All of the animals of ACLT surgery developed OA that was evidenced by histology and micro-CT.

### Gross Morphological Examination

**Figure 2:**
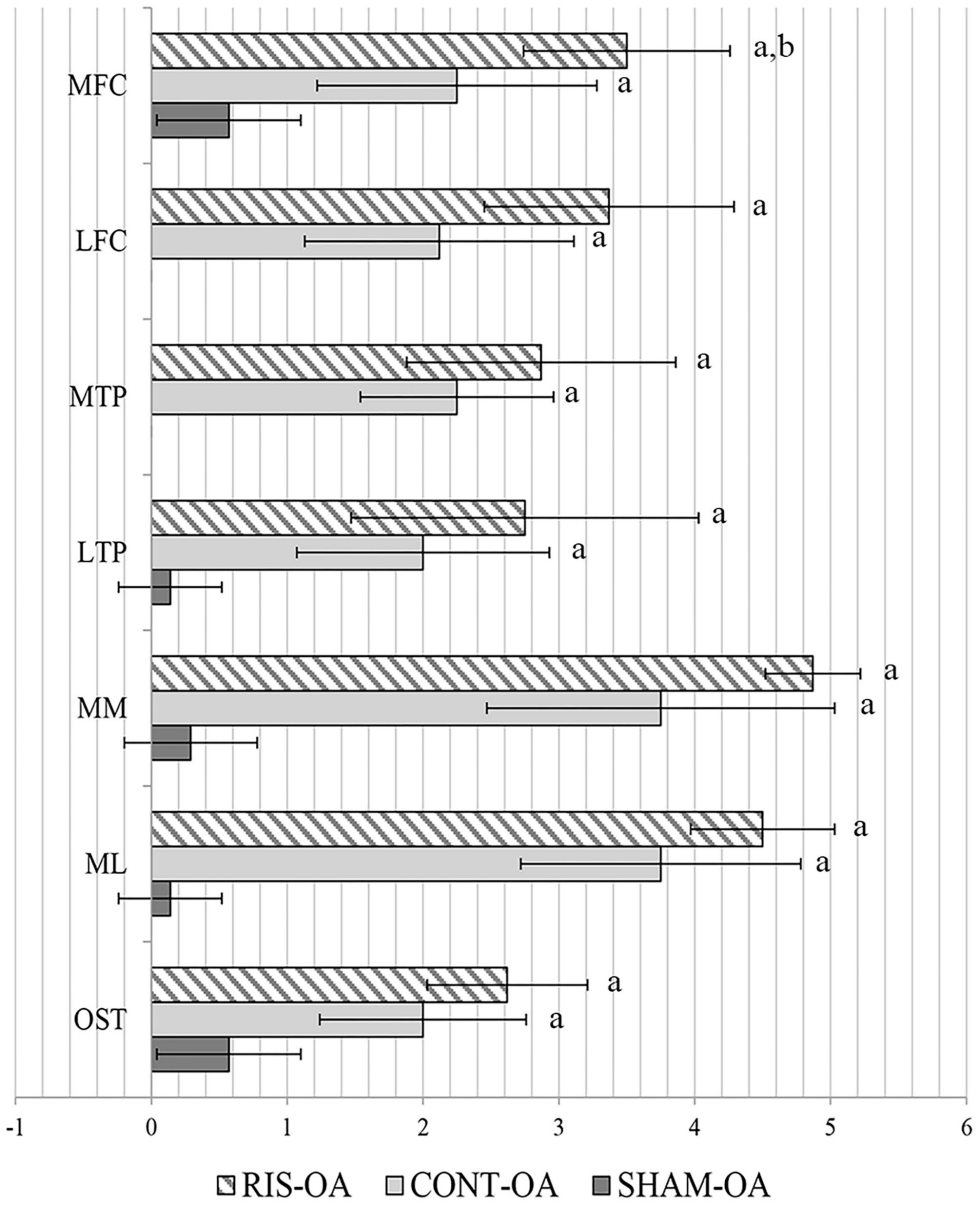
Macroscopic assessment of OA joints. MFC, medial femoral condyle; LFC, lateral femoral condyle; MTP, medial tibial plateau; LTP, lateral tibial plateau; MM, medial meniscus; ML, lateral meniscus; OST, osteophytes. Statistical differences *p* < 0.05: ^a^vs. SHAM, ^b^vs. CONT.

The comparison scores are available on Supplementary Table 1. Macroscopic evaluation revealed typical images of OA disease caused by ACLT and partial meniscectomy surgery, including rougher cartilage surface and ulcerations, meniscal damage, and osteophyte formation. The stifle OA joints studied showed significant differences between the simulated surgery group (SHAM-OA) against the control group (CONT-OA) and the risedronate treated-group (RIS-OA). No significant differences were found in terms of severity of lesions between the animals treated with risedronate and control animals, except for the values obtained in the medial femoral condyle, where the risedronate-treated group presented a significantly increased macroscopic lesions compared to the control group (*p* = 0.006). The highest scores of OA severity were observed in the group treated with oral risedronate for all parameters evaluated. Generally, the changes observed in the medial compartment, where surgery was performed, were slighty more pronounced than those in the lateral compartment. However, no statistical differences were found. The macroscopic grades of the articular surface of OA stifle joints are shown in Figure 2. Regarding the healthy joints (HT), no significant differences were found in any of the studied groups. All of them were graded as clinically normal showing a smooth cartilage surface in femoral and tibial compartments and absence of osteophytes.

### Histology Qualitative Results

**Figure 3:**
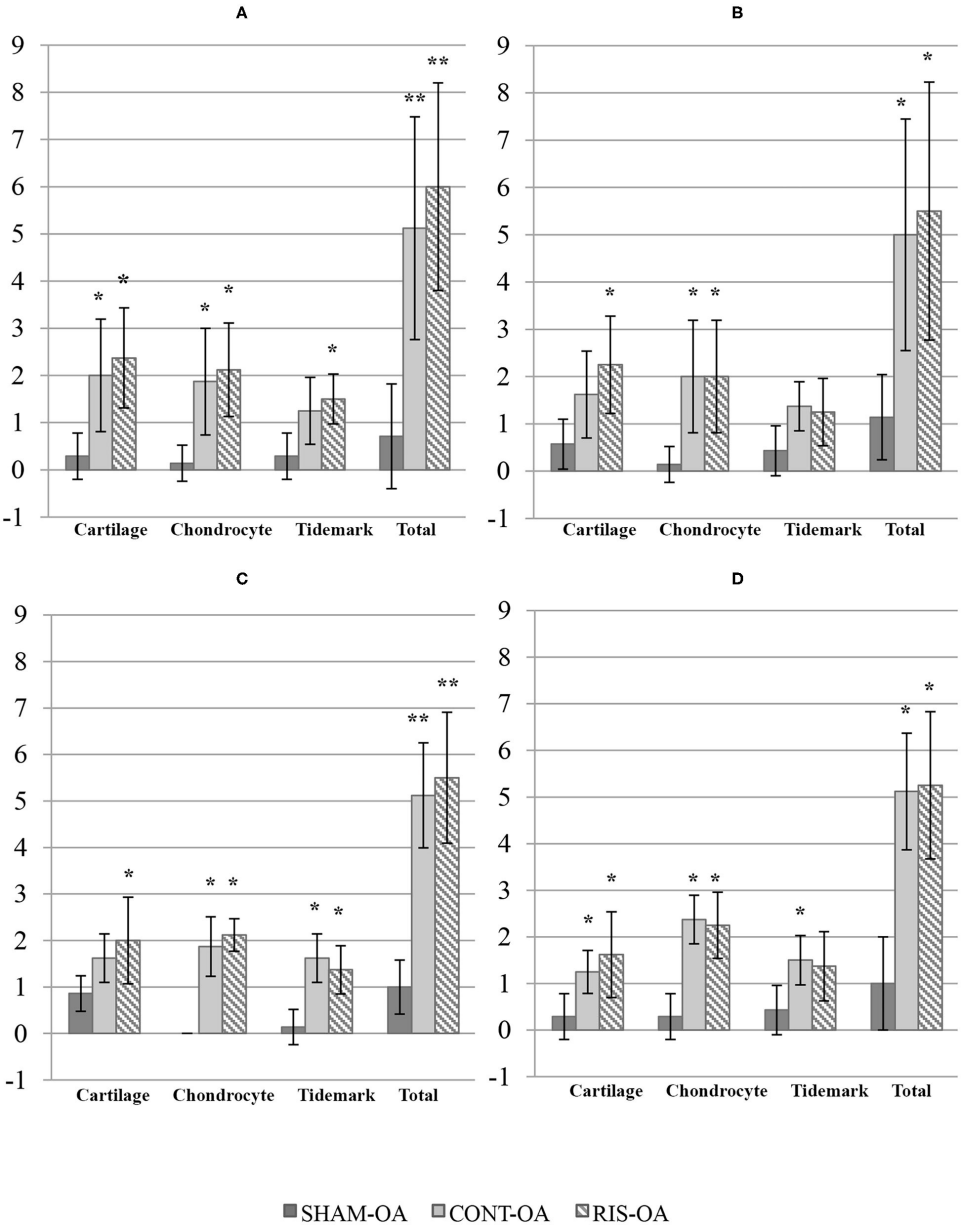
Histologic scores for microscopic cartilage alterations on OA stifle joints. **(A)** Medial femoral condyle; **(B)** Lateral femoral condyle; **(C)** Medial tibial plateau; **(D)** Lateral tibial plateau. Values given as mean ± SD. Statistical differences vs. SHAM: *p < 0.05, **p < 0.001.

**Figure 4:**
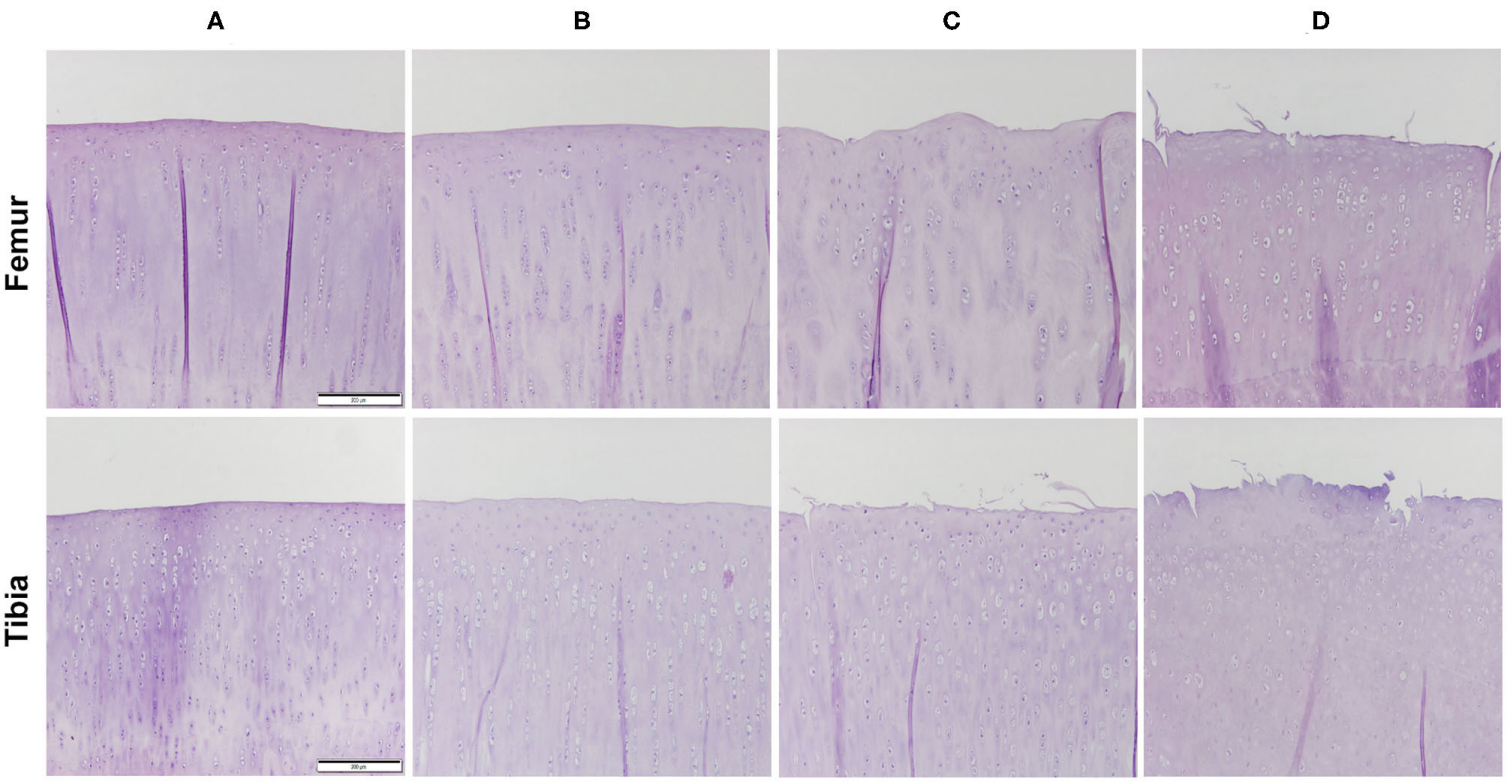
Representative histology images of the articular cartilage stained with hematoxylin and eosin (H-E). Magnification 10X. **(A)** HT; **(B)** SHAM-OA; **(C)** CONT-OA; **(D)** RIS-OA.

Histologic scores for microscopic cartilage and synovial alterations are shown in Supplementary Table 2. Regarding the severity of cartilage pathology, the highest scores were found in medial femoral condyle, but no statistical differences were found based on the compartment studied. In relation to tidemark integrity, it was the most variable parameter and in some instances, no differences were found even compared to the SHAM group. Total histologic cartilage evaluation showed significantly higher scores in the CONT-OA and RIS-OA groups than in the SHAM-OA group. Nevertheless, no differences were found when comparing control and risedronate-treated OA groups. The mean scores and SD of the parameters evaluated for OA stifle joints are shown in Figure 3. As observed in the macroscopic evaluation, slightly higher values of osteoarthritis were determined in RIS-OA group. In summary, treatment with risedronate did not show any histologic signs of improvement on the cartilage structure compared to the placebo treated rabbits with knee surgical injury (Figure 4). Regarding healthy joints, all showed smooth cartilage surface, normal chondrocyte cellularity and intact tidemark. No significant differences were found in any of the compared groups, even between SHAM-OA, and SHAM-HT animals.

**Figure 5:**
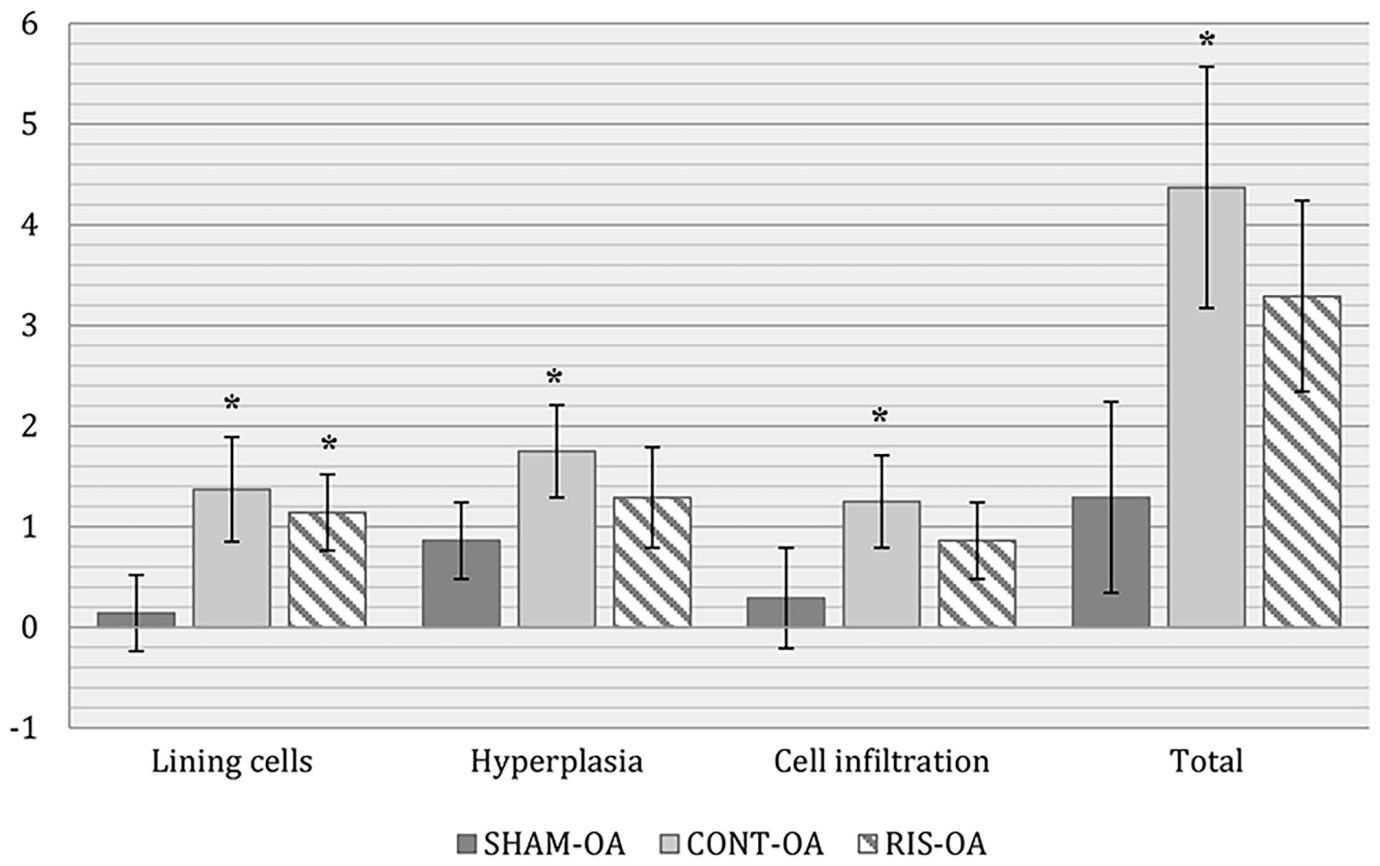
Histologic scores for microscopic synovial changes on OA stifle joints. Values given as mean ± SD. Statistical differences vs. SHAM: *p < 0.05.

**Figure 6:**
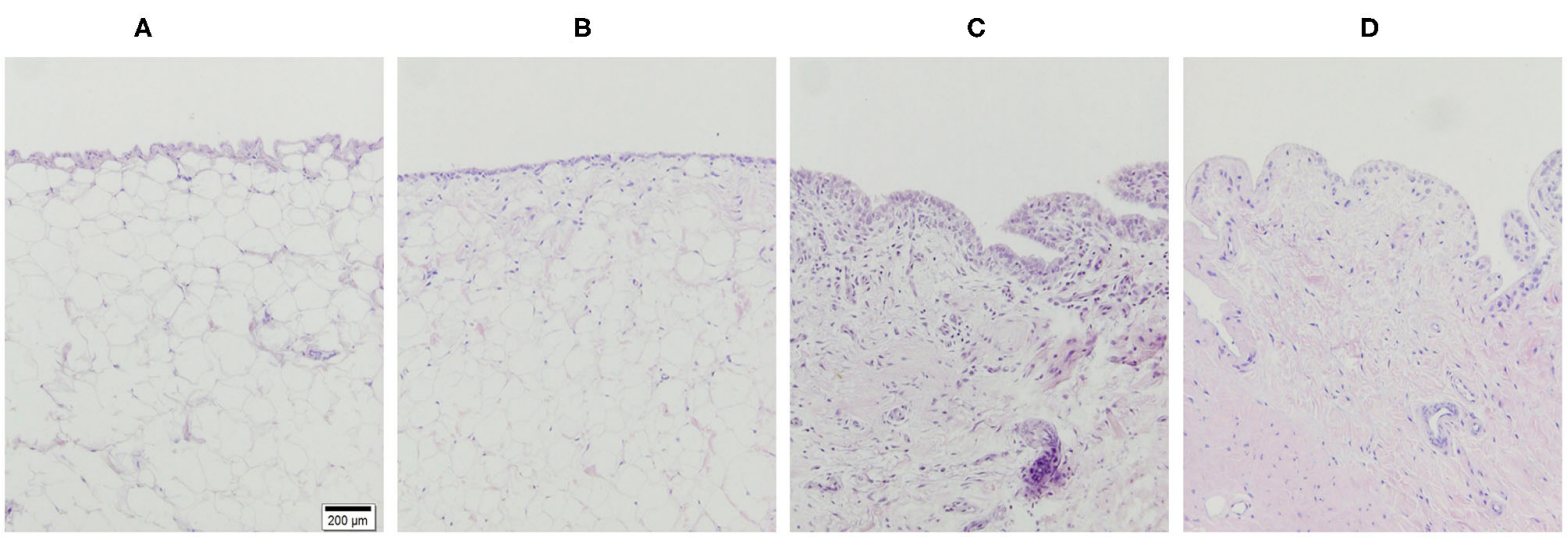
Representative histology images of the synovial membrane stained with H-E. Magnification 10X. **(A)** HT; **(B)** SHAM-OA; **(C)** CONT-OA; **(D)** RIS-OA.

In the synovial membrane, significant differences were detected between the SHAM-OA and the CONT-OA groups in the three variables measured. Risedronate treatment group (RIS-OA) presented scores closer to SHAM-OA samples and the results were slightly less pronounced than those of the control group (Figure 5). Regarding the results observed in healthy knees, no significant differences were found in any of the parameters analyzed. As expected, healthy joints did not show inflammatory infiltrations or villous hyperplasia and the lining surface was thin (Figure 6).

### Micro-CT Results

Results can be summarized in three points:

#### Osteophyte Formation

**Figure 7:**
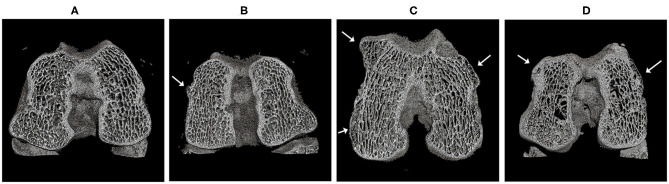
Micro-CT scan and three-dimensional reconstruction of the femur samples with representative osteophyte development. **(A)** HT; **(B)** SHAM-OA; **(C)** CONT-OA; **(D)** RIS-OA. Osteophytes were indicated by arrows.

No osteophyte formation was identified on healthy limbs. However, all osteoarthritic joints exhibited osteophyte development, although, the scores in SHAM-OA group were significantly lower. Regarding the total volume of femur osteophytes, statistical differences were observed compared to the SHAM-OA group. Despite the fact that the untreated injured limbs developed the highest values, no significant differences were found between CONT-OA and RIS-OA. Although injured-knees showed the highest mineralization values, there were no statistical differences in microstructural parameters in terms of bone volume fraction (BV/TV) or in volumetric bone mineral density (vBMD). Risedronate treatment does not seem to reduce bony osteophyte development in OA joints (Table 2 and Figure 7).

**Table 2:** Volumetric micro-CT measurements of osteophyte formation in osteoarthritic femurs.

	**SHAM-OA**	**CONT-OA**	**RIS-OA**
**TV (mm^3^)**	7.03 ± 11.63	**38.59 ± 19.58^a^**	**34.21 ± 19.52^a^**
**BV/TV (%)**	23.42 ± 29.28	49.82 ± 9.64	58.34 ± 10.37
**vBMD (mg/cm^3^)**	219.90 ± 275.34	407.93 ± 168.02	539.69 ± 184.69

*TV, Total volume; BV/TV, Bone volume fraccion; vBMD, Volumetric bone mineral density. Values represent the mean and SD. Statistical significant differences are marked in “bold text.” p < 0.05*:

a *vs. SHAM*.

#### Subchondral Bone Plate Thickness

**Figure 8:**
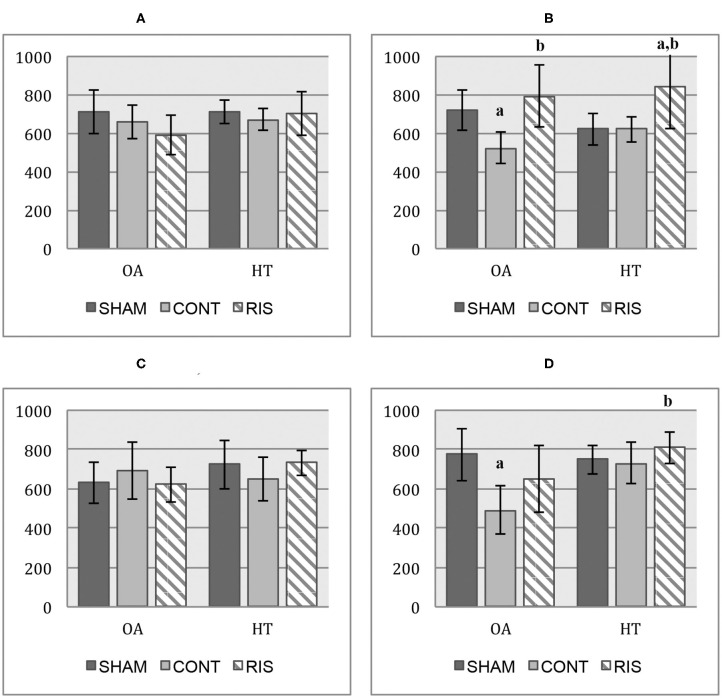
Subchondral bone plate thickness micro-CT values (μm). **(A)** medial femoral condyle; **(B)** lateral femoral condyle; **(C)** medial tibial plateau; **(D)** lateral tibial plateau. Values given as mean ± SD. Statistical differences *p* < 0.05: ^a^vs. SHAM, ^b^vs. CONT.

In the evaluation of subchondral bone plate thickness, no noticeable differences were found in medial compartments. However, in lateral compartments, risedronate-treated groups showed a tendency to increase the values of the subchondral bone plate thickness in both OA and healthy joints (Figure 8).

#### Trabecular Parameters

Regarding the trabecular parameters, the main statistical differences were found between the RIS-OA group and the SHAM-OA group when evaluating vBMD, BV/TV, Tb.Sp, and Tb.N. The results obtained in the osteoarthritic groups (CONT-OA and RIS-OA) showed that vBMD tended to be lower in injured limbs with significant differences compared to SHAM-OA, in all the compartments evaluated. In relation to BV/TV, the injured limbs showed a tendency of decreased values, but the differences when comparing CONT-OA and RIS-OA were not significant. Trabecular separation was increased in RIS-OA and RIS-HT animals, finding significant differences when comparing them to the SHAM groups. Regarding the trabecular number measurement, RIS-OA animals presented decreased values, and this difference was significant when compared to the SHAM-OA group (*p* = 0.003) and even to the CONT-OA group in the medial femur condyle (*p* = 0.016). In the case of healthy joints, as observed in OA samples, risedronate-treated animals showed a significant Tb.N reduction. Micro-CT did not reveal significantly reduced levels of trabecular bone loss in knee-injured animals treated with risedronate compared to untreated animals. Micro-CT trabecular data are shown in Table 3.

**Table 3:** Micro-CT analysis.

			**vBMD (mg/cm^**3**^)**	**BV/TV (%)**	**Tb.Th (mm)**	**Tb.Sp (mm)**	**Tb.N (mm^**−1**^)**	**Tb.Pf (mm^**−1**^)**
**Osteoarthritis joints (OA)**	**SHAM**	**LFC**	695.43 ± 116.60	62.37 ± 4.56	0.16 ± 0.02	0.10 ± 0.01	3.89 ± 0.24	3.03 ± 1.46
		**MFC**	708.81 ± 95.65	63.62 ± 3.52	0.16 ± 0.02	0.10 ± 0.02	3.94 ± 0.34	1.92 ± 2.97
		**LTP**	735.11 ± 106.36	61.92 ± 13.99	0.14 ± 0.04	0.10 ± 0.03	4.61 ± 0.42	−0.86 ± 4.89
		**MTP**	709.86 ± 98.55	59.20 ± 12.84	0.15 ± 0.04	0.11± 0.04	4.08 ± 0.71	3.66 ± 4.73
	**CONT**	**LFC**	**452.39 ± 158.99^a^**	**50.17 ± 8.59^a^**	0.14 ± 0.02	0.15 ± 0.06	3.60 ± 0.46	5.44 ± 2.22
		**MFC**	**546.15 ± 134.36^a^**	**55.94 ± 6.50^a^**	0.15 ± 0.02	0.13 ± 0.04	3.75 ± 0.39	3.89 ± 2.98
		**LTP**	**487.01 ± 108.37^a^**	52.67 ± 6.46	0.12 ± 0.02	0.12 ± 0.03	4.29 ± 0.55	3.39 ± 2.57
		**MTP**	**537.37 ± 109.40^a^**	55.81 ± 6.01	0.14 ± 0.02	0.14 ± 0.06	3.97 ± 0.60	3.64 ± 3.15
	**RIS**	**LFC**	**457.84 ± 170.76^a^**	**50.40 ± 10.02^a^**	0.16 ± 0.02	**0.18 ± 0.05^a^**	**3.22 ± 0.43^a^**	3.81 ± 4.58
		**MFC**	**456.73 ± 125.69^a^**	**50.31 ± 6.47^a^**	0.16 ± 0.02	**0.18 ± 0.04^a^**	**3.23 ± 0.32^a,b^**	3.81 ± 2.63
		**LTP**	**449.35 ± 226.52^a^**	53.23 ± 9.64	0.14 ± 0.03	**0.14 ± 0.04^a^**	**3.84 ± 0.50^a^**	2.15 ± 3.74
		**MTP**	**446.98 ± 129.22^a^**	**47.80 ± 9.30^a^**	0.15 ± 0.34	**0.21 ± 0.08^a^**	**3.22 ± 0.59^a^**	4.47 ± 3.57
**Healthy joints (HT)**	**SHAM**	**LFC**	821.91 ± 74.53	67.07 ± 2.54	0.17 ± 0.01	0.08 ± 0.01	3.85 ± 0.18	2.52 ± 1.35
		**MFC**	861.11 ± 58.66	69.84 ± 1.55	0.18 ± 0.01	0.08 ± 0.01	3.94 ± 0.14	−0.69 ± 1.41
		**LTP**	758.38 ± 185.16	69.23 ± 8.73	0.15 ± 0.03	0.08 ± 0.02	4.72 ± 0.80	−4.48 ±7.01
		**MTP**	813.52 ± 82.51	63.30 ± 10.88	0.15 ± 0.04	0.09 ± 0.04	4.28 ± 0.85	0.29 ± 5.16
	**CONT**	**LFC**	773.16 ± 86.54	65.98 ± 3.34	0.17 ± 0.02	0.09 ± 0.02	3.84 ± 0.39	1.26 ± 1.71
		**MFC**	806.95 ± 101.34	67.77 ± 4.36	0.18 ± 0.02	0.10 ± 0.01	3.73 ± 0.38	0.46 ± 2.44
		**LTP**	758.40 ± 227.16	59.06 ± 18.72	0.14 ± 0.05	0.11 ± 0.05	4.41 ± 0.44	−0.66 ± 7.21
		**MTP**	666.73 ± 231.44	53.37 ± 16.43	0.15 ± 0.06	0.15 ± 0.07	3.82 ± 0.61	3.75 ± 6.29
	**RIS**	**LFC**	758.16 ± 66.75	64.04 ± 3.09	0.19 ± 0.01	**0.11 ± 0.01^a^**	**3.33 ± 0.20^a,b^**	**4.45 ± 2.47^b^**
		**MFC**	749.07 ± 94.66	64.11 ± 4.34	0.19 ± 0.01	**0.11 ± 0.02^a,b^**	**3.39 ± 0.26^a^**	**3.54 ± 3.34^a^**
		**LTP**	733.36 ± 171.25	61.67 ± 14.99	0.16 ± 0.05	**0.12 ± 0.06^a^**	**4.02 ± 0.39^a^**	−1.00 ± 3.40
		**MTP**	688.92 ± 87.87	54.36 ± 12.42	0.17 ± 0.05	0.17 ± 0.09	3.44± 0.77	4.16 ± 6.43

*Micro-CT results: vBMD, volumetric bone mineral density; BV/TV, bone volume fraccion; Tb.Th, trabecular thickness; Tb:Sp, trabecular separation; Tb:N, trabecular number; Tb.Pf, trabecular pattern factor. Values represent the mean and SD. Statistical significant differences are marked in “bold text.” p < 0.05*:

a *vs. SHAM*,

b *vs. CONT*.

## Discussion

The present study analyzed the effect of risedronate on subchondral trabecular bone microarchitecture, bone turnover, and cartilage degeneration in a long-term experimental rabbit knee model of ACLT and partial medial meniscectomy. Animal preclinical models of post-traumatic OA may provide an excellent possibility to resolve the actual controversy of how to evaluate the effect of different drugs on the progress of the disease, allowing us to assess the direct effect of their administration in joint tissues. Nevertheless, they do not fully reproduce the complex etiology developed in humans and the results cannot be totally comparable (14, 34). With respect to the surgically induced methods described, ACLT is one of the most common, and is well-established. This technique leads to a gradual instability with a progressive degeneration, similar to that observed in clinical OA. Rabbit ACLT models are increasingly used in OA studies, demonstrating a fast damage of the cartilage, and intermediate subchondral bone changes (32, 33, 44).

Alterations in articular cartilage surface, such as cartilage fibrillation and full-thickness erosion were easily detected through macroscopic and histologic techniques, which provide a complete assessment of cartilage status. Nevertheless, they currently only provide qualitative or semi-quantitative information and sometimes there may be difficulties regarding their validity and reproducibility (41, 44, 45). That is the reason why other advanced 3D imaging techniques such as micro-CT have been successfully implemented in animal models of OA. Micro-CT can be used to accurately quantify cartilage damage and calcified tissue changes in the ACLT model of OA (36, 46, 47).

In spite of the high prevalence of OA in human and veterinary medicine, at present, there is little to offer to affected individuals for the prevention of the disease or treatment in the early stages (48). In this regard, antiresorptive agents have been proposed as potential disease-modifying OA drugs. It is believed that bisphosphonates could modulate the subchondral bone remodeling and consequently inducing changes in the OA progression, reducing the cartilage degeneration (3, 10, 13, 18–20, 49). In previous preclinical studies, risedronate has shown a reduction in the cartilage pathology (50) and changes in cancellous bone microarchitecture leading to improved mechanical properties (17, 23, 24). However, contradictory results in clinical trials hindered their acceptation regarding the disease-modifying efficacy in the scientific community (15, 51). As an attempt to clarify their efficacious in human knee osteoarthritis some systematic reviews were conducted concluding that, the risedronate effects on inflammation relief, and pain control seems to be limited (52, 53).

In this study, our objective was to evaluate the effect of risedronate on the progression of post-traumatic OA. To achieve it, we used qualitative macroscopic and histological assessments of articular cartilage knee and synovial membrane in non-calcified samples, as well as, a high-resolution micro-CT scanner to evaluate the three-dimensional microstructure and volume of subchondral bone plate, trabecular bone, and osteophyte formation. Risedronate have been extensively studied in rabbit preclinical models in order to evaluate their structural and osteogenic effects as antiresorptive agents (54–58). Additionally, as previously reported, their effects against osteoarthritic changes on synovial joint tissues were also analyzed in this animal model (23–26). However to the best of our knowledge, no previous OA research studies have analyzed the influence of long-term risedronate administration on cartilage and subchondral bone in a rabbit model of OA.

As previously published, in the macroscopic evaluation of knee joint samples, we noted that the medial compartments showed slightly greater degeneration than the lateral compartments. In addition, femur also demonstrated a slightly greater deterioration than that observed in tibias (42, 44). Even though some authors observed that administration of BPs showed a significant improvement in the cartilage surface and a minor number of osteophytes (3, 59), in our study, risedronate did not show any improvement in the cartilage condition and did not supress the osteophyte formation.

In our histological section, moderate to severe OA lesions were observed in the operated limbs. Specifically, cartilage assessment in non-calcified samples proved that risedronate treatment, over long periods of time, was not able to reduce the cartilage damage, showing similar or even greater degenerative changes compared to the untreated rabbits. This supports the findings of other preclinical studies, which did not find any chondroprotective effect of BP treatment on articular cartilage damage score either (12, 14). On the other hand, some authors reported a significant positive effect of bisphosphonates on OA severity scores, showing fewer degenerative changes compared to the untreated animals (3, 10, 13, 17, 19, 49, 50). Moreover, a previous study of our research group, has shown a modest chondroprotective effect with short-term risedronate administration (24). Regarding histological synovial membrane assessment, we observed that risedronate administration did not present a significant efficacy in the pathological changes evaluated. However, we noticed a slightly anti-inflammatory activity as previously published in early stages of OA (24, 60).

As far as osteophytogenesis is concerned, several authors tested the efficacy of the risedronate treatment in the inhibition of osteophyte formation (15, 17, 25). In accordance with the findings of other groups (25), we showed that risedronate did not positively suppress or delay osteophytosis. Moreover, RIS did not inhibit osteophyte maturation compared to untreated animals. In the case of other used BPs, such as alendronate, it has been reported to be effective in reducing osteophyte development (3, 17, 59). This seems to be related to a reduced transforming growth factor β (TGF- β), as a possible osteophyte inhibitor. However, although risedronate has also been shown to reduce TGF- β, strangely, it does not seem to influence the osteophyte suppression (3, 17, 25). In our study, all the injured limbs exhibited osteophyte formation, including the SHAM-OA group. Although the osteophyte severity scores were really low in this group, one should also note that it is the only articular exposure that seems to be able to induce the osteophyte formation. In our opinion, this point is interesting, because in some situations, clinical practices considered minimally invasive, such as arthrocentesis or arthroscopies, could predispose to secondary OA.

In addition to the degenerative damage shown in cartilage, during OA progression, bone adaptations were described, such a progressive subchondral bone sclerosis, and it was strongly correlated with subchondral bone plate thickness (4, 11, 61). The association between cartilage degeneration and subchondral bone plate thickness did not appear to be unequivocal, and conflicting results were found in this case (21). Some studies identified significant increases in the bone plate thickness with bisphosphonates treatment (17, 19, 21, 59) and others, did not find any differences between control and treatment groups (13). The same as Thomsen et al. (12), we observed that risedronate administration for 24 weeks showed a slight tendency to increase subchondral bone plate thickness, compared to that of untreated animals. In our case, this was mainly observed in lateral compartments of operated and non-operated joints. This could indicate an altered bone remodeling caused by the effects of the bisphosphonate drug. However, these changes did not seem to result in an articular cartilage damage reduction and they may even, reduce the ability to dissipate the load on articular cartilage, increasing OA severity in unstable joints.

The extent of the influence of BPs treatments on subchondral bone and articular cartilage damage is still unclear. Shirai et al. (13) showed the chondroprotective effect of alendronate, supporting the hypothesis that it prevented periarticular bone lose and might also reduce cartilage erosion (3, 62). On the contrary, Ding et al. (21) observed that alendronate did not have a chondroprotective effect on the cartilage degeneration and suggested that the increased subchondral bone mass and density, observed after BP administration, could cause OA disease progression promoting cartilage stress in load bearing.

Consistent with the results of other research studies, alterations were detected as soon as 3 weeks after surgery. The main changes observed in the trabecular bone architecture were: decreased bone mineral density (BMD), reduced bone volume ratio (BV/TV) and smaller trabecular thickness (Tb.Th) (44, 46, 63). In a previous study, we have reported that no signs of advanced OA disease were shown in subchondral bone at 11 weeks after surgery (24). However, Boyd et al. (46) supported the presence of microstructural changes on the periarticular cancellous bone in early stages of the disease. Although, results could be different depending on when the evaluation was carried out and on the surgical procedure selected (64), the main tendency in the evolution of the disease being an increased deterioration in the quality of the trabecular bone (47). Some authors observed an improved trabecular bone volume, number, and thickness with BP administration compared to those of untreated groups (13, 14, 19, 23, 65). In the specific case of risedronate, a positive effect was noted, preserving subchondral bone properties in short-term administrations in a rabbit model of OA (23, 25, 60). Nevertheless, in our case, risedronate administration did not seem to preserve trabecular bone mass, exhibited decrease in overall bone quality with loss of vBMD, a tendency to decrease BV/TV and Tb.N, and increase Tb.Sp. We should especially point out the small differences that were found between RIS-OA and CONT-OA groups in most of the analyzed values. These findings suggest that long-term risedronate therapy may not have a beneficial effect on subchondral trabecular bone microarchitecture compared to the control (placebo administration) group.

The disuse of the operated limb may have caused this bone reduction (21). However, we also should consider that these degenerative changes can be quite variable among different species and individuals in the same group, indicating the possible presence of biologic differences in the surgical animal model (44). Although the animal physical activity in laboratories is quite limited, weigh-bearing shifts to healthy joints are typical behaviors shown by rabbits, in order to reduce pain (14). On that basis, we used contralateral joints as healthy controls. In agreement with other publications, classical signs of OA disease were not observed in these samples, suggesting the validity of the use of the contralateral joint as an acceptable healthy test (8, 46). Another advantage is that inter-animal differences may be reduced when we use the opposite limb (63). Irrespective of that point, the inclusion of the SHAM group as a control surgery group could also provide additional information regarding the possibly altered limb-loading patterns or compensatory locomotion after ACLT. In our study, in most of the evaluated parameters, no significant statistical differences were found between healthy control groups and neither between the SHAM-HT and SHAM-OA groups. However, slightly higher OA damage values were observed in the latter. As mentioned previously, this could show that the simple joint articular capsule exposure may predispose to initial OA disease development.

According to previous publications, it seems that early bisphosphonate therapy may help to preserve periarticular bone properties, slowing down the short-term degeneration (10, 23, 26). In OA pathology, histological changes are time-dependent (42) thus, the time point of treatment initiation is crucial for treating OA and similarly, the efficacy of BPs depends on the disease stage (10, 62). In this regard, a more beneficial effect with preventive treatment administration was observed (3). Nevertheless, when articular cartilage is damaged, antiresorptive therapies do not seem to be effective (27). The rabbits used in our study received risedronate treatment only 3 weeks after surgery, and despite that, antiresorptive therapy failed to maintain cartilage health and prevent bone loss and osteophyte formation, as suggested by other authors (23).

Presumably, this lack of consensus regarding the administration of risedronate and its disease-modifying capacity could be associated with oral bioavailability, stage of OA in experimental groups, use of different experimental animal models and dosage of treatments administered. Furthermore, the different evaluation methods make direct comparisons among these studies challenging. It is possible that higher doses of BPs than those used in osteoporosis treatments may be necessary in order to achieve the inhibition of cartilage degradation and osteophyte formation, and may have some benefit in late-stage OA (3, 7, 65). Regarding the experimental time frame of the therapy administration, most studies are based on short periods of time. For this reason, further clinical studies are needed to delve into and validate these findings, as a critical step toward understanding the pathophysiology of the disease in advanced stages, as well as to evaluate the effect of therapeutic interventions on the joint tissue.

This study provides novel information about the effect of long-term risedronate use on cartilage and subchondral bone changes in a rabbit model of OA and contributes to better understanding the disease mechanisms in advanced stages of OA.

As limitations, the small number of samples per group consistently hinders the detection of significant effects. Although we standardized the regions of interest for the analysis to the extent possible, small variability differences may be shown in the measurements. Another limitation of the present study is the use of *ex vivo* techniques for the description of degenerative joint lesions and the results may not be completely extrapolated to live animals. Lastly, we took measurements only once, 27 weeks after surgery (endpoint of study).

## Conclusions

Long-term risedronate administration did not demonstrate a chondroprotective effect, showing similar or even greater degenerative changes compared to those of untreated animals. Regarding the histological synovial membrane assessment, a slight improvement in inflammatory changes was noticeable. Risedronate treatment appeared to increase subchondral bone plate thickness in lateral compartments in both OA and healthy joints. However, neither subchondral bone quality enhancement was evident, nor was it able to inhibit the osteophyte development in OA joints. Consequently, oral risedronate treatment did not have the capacity to prevent the osteoarthritis progression in a rabbit instability model of OA after 6 months of treatment. The present results suggest that it seems unlikely that risedronate could be effective as a disease-modifying osteoarthritic drug.

## Data Availability Statement

The original contributions presented in the study are included in the article/Supplementary Material, further inquiries can be directed to the corresponding author/s.

## Ethics Statement

The animal study was reviewed and approved by Ethical Committee of the University of Santiago de Compostela (Reference number: 01/16/LU-002).

## Author Contributions

All authors undertook the experimental work. ML-P, FM, and AG-C conceived the concept for the paper and designed the study. Micro-CT assessments were made by MP, and histological analyses were performed by SF-M, ML-P, and FM. All authors provided critical input and collaborated on data analysis, interpretation of results, contributed to the writing of the manuscript, and have approved the final version.

## Conflict of Interest

The authors declare that the research was conducted in the absence of any commercial or financial relationships that could be construed as a potential conflict of interest.
