# Supplementary material for: No Effect of Long-Term Risedronate Use on Cartilage and Subchondral Bone in an Experimental Rabbit Model of Osteoarthritis
Source: Front Vet Sci. 2020 Nov 2;7:576212. doi: 10.3389/fvets.2020.576212 (PMC7667022; doi:10.3389/fvets.2020.576212)
Supplement: Supplementary file 1 [file Table_1.pdf]

Supplementary Table 1. Gross morphological examination of OA and healthy joints.

MACROSCOPIC GRADING						
	**SHAM-OA**	**CONT-OA**	**RIS-OA**	**SHAM-HT**	**CONT-HT**	**RIS-HT**
**MFC**	0.57 ± 0.53	**2.25 ± 1.03**<br>$^{a}p = 0.001$	**3.50 ± 0.76**<br>$^{a}p < 0.001$<br>$^{b}p = 0.006$	0.57 ± 0.52	0.37 ± 0.59	0.62 ± 0.59
**LFC**	0.00 ± 0.00	**2.12 ± 0.99**<br>$^{a}p < 0.05$	**3.37 ± 0.92**<br>$^{a}p < 0.05$	0.00 ± 0.00	0.12 ± 0.35	0.37 ± 0.59
**MTP**	0.00 ± 0.00	**2.25 ± 0.71**<br>$^{a}p < 0.05$	**2.87 ± 0.99**<br>$^{a}p < 0.05$	0.43 ± 0.53	0.12 ± 0.35	0.62 ± 0.59
**LTP**	0.14 ± 0.38	**2.00 ± 0.93**<br>$^{a}p = 0.003$	**2.75 ± 1.28**<br>$^{a}p < 0.001$	0.29 ± 0.49	0.37 ± 0.59	0.12 ± 0.35
**MM**	0.29 ± 0.49	**3.75 ± 1.28**<br>$^{a}p < 0.05$	**4.87 ± 0.35**<br>$^{a}p < 0.05$	1.14 ± 0.38	1.12 ± 0.35	1.50 ± 0.53
**LM**	0.14 ± 0.38	**3.75 ± 1.03**<br>$^{a}p < 0.001$	**4.50 ± 0.53**<br>$^{a}p < 0,001$	1.14 ± 1.07	1.62 ± 0.52	0.87 ± 0.35
**OST**	0.57 ± 0.53	**2.00 ± 0.76**<br>$^{a}p < 0.05$	**2.62 ± 0.59**<br>$^{a}p < 0.05$	0.00 ± 0.00	0.00 ± 0.00	0.25 ± 0.46

MFC: medial femoral condyle; LFC: lateral femoral condyle; MTP: medial tibial plateau; LTP: lateral tibial plateau; MM: medial meniscus; LM: lateral meniscus; OST: osteophytes. The values are mean ± SD. Statistical differences $p< 0.05$: ^{a} vs. SHAM, ^{b} vs. CONT.
