# Supplementary material for: No Effect of Long-Term Risedronate Use on Cartilage and Subchondral Bone in an Experimental Rabbit Model of Osteoarthritis
Source: Front Vet Sci. 2020 Nov 2;7:576212. doi: 10.3389/fvets.2020.576212 (PMC7667022; doi:10.3389/fvets.2020.576212)
Supplement: Supplementary file 2 [file Table_2.pdf]

Supplementary Table 2. Histology qualitative results.

			SHAM-OA	CONT-OA	RIS-OA	SHAM-HT	CONT-HT	RIS-HT
CARTILAGE	MFC	**Cartilage pathology**	0.29 ± 0.49	**2.00 ± 1.19 ***	**2.37 ± 1.06 ***	0.43 ± 0.53	0.75 ± 0.71	0.12 ± 0.35
		**Chondrocyte pathology**	0.14 ± 0.38	**1.87 ± 1.13 ***	**2.12 ± 0.99 ***	0.14 ± 0.38	0.75 ± 0.71	0.50 ± 0.53
		**Tidemark**	0.29 ± 0.49	1.25 ± 0.71	**1.50 ± 0.53 ***	0.14 ± 0.38	0.62 ± 0.52	0.50 ± 0.53
		**Total**	0.71 ± 1.11	**5.12 ± 2.36 ****	**6.00 ± 2.20 ****	0.71 ± 0.49	2.12 ± 1.64	1.12 ± 1.13
	LFC	**Cartilage pathology**	0.57 ± 0.53	1.62 ± 0.92	**2.25 ± 1.03 ***	0.29 ± 0.49	0.71 ± 0.49	0.50 ± 0.53
		**Chondrocyte pathology**	0.14 ± 0.38	**2.00 ± 1.19 ****	**2.00 ± 1.19 ***	0.14 ± 0.38	0.71 ± 0.76	0.50 ± 0.53
		**Tidemark**	0.43 ± 0.53	1.37 ± 0.52	1.25 ± 0.71	0.14 ± 0.38	0.57 ± 0.53	0.62 ± 0.52
		**Total**	1.14 ± 0.90	**5.00 ± 2.45 ****	**5.50 ± 2.73 ***	0.57 ± 0.79	2.00 ± 1.63	1.62 ± 1.06
	MTP	**Cartilage pathology**	0.86 ± 0.38	1.62 ± 0.52	**2.00 ± 0.93 ***	0.43 ± 0.53	0.75 ± 0.46	0.57 ± 0.53
		**Chondrocyte pathology**	0.00 ± 0.00	**1.87 ± 0.64 ***	**2.12 ± 0.35 ***	0.00 ± 0.00	0.00 ± 0.00	0.43 ± 0.53
		**Tidemark**	0.14 ± 0.38	**1.62 ± 0.52 ***	**1.37 ± 0.52 ***	0.14 ± 0.38	0.37 ± 0.52	0.43 ± 0.53
		**Total**	1.00 ± 0.58	**5.12 ± 1.13 ****	**5.50 ± 1.41 ****	0.57 ± 0.53	1.12 ± 0.83	1.43 ± 0.98
	LTP	**Cartilage pathology**	0.29 ± 0.49	**1.25 ± 0.46 ***	**1.62 ± 0.92 ***	0.00 ± 0.00	0.37 ± 0.52	0.40 ± 0.55
		**Chondrocyte pathology**	0.29 ± 0.49	**2.37 ± 0.52 ***	**2.25 ± 0.71 ***	0.14 ± 0.38	0.12 ± 0.35	0.20 ± 0.45
		**Tidemark**	0.43 ± 0.53	**1.50 ± 0.53 ***	1.37 ± 0.74	0.00 ± 0.00	0.50 ± 0.53	0.60 ± 0.55
		**Total**	1.00 ± 1.00	**5.12 ± 1.25 ***	**5.25 ± 1.58 ***	0.14 ± 0.38	1.00 ± 0.53	1.20 ± 1.09
SYNOVIAL	**Lining cell characteristics**		0.14 ± 0.38	**1.37 ± 0.52 ***	**1.14 ± 0.38 ***	0.00 ± 0.00	0.00 ± 0.00	0.00 ± 0.00
	**Hyperplasia**		0.86 ± 0.38	**1.75 ± 0.46 ***	1.28 ± 0.49	0.43 ± 0.53	0.50 ± 0.53	0.43 ± 0.53
	**Cell infiltration characteristics**		0.29 ± 0.49	**1.25 ± 0.46** *	0.86 ± 0.38	0.00 ± 0.00	0.00 ± 0.00	0.14 ± 0.38
	**Total**		1.29 ± 0.95	**4.37 ± 1.19 ***	3.29 ± 0.95	0.43 ± 0.53	0.50 ± 0.53	0.57 ± 0.79

MFC: medial femoral condyle; LFC: lateral femoral condyle; MTP: medial tibial plateau; LTP: lateral tibial plateau. The values are mean ± SD. Statistical differences vs. SHAM: * $p< 0.05$, ** $p < 0.001$
